# Epsom Salts for the Pain in Burns

**Published:** 1894-03

**Authors:** N. F. Howard

**Affiliations:** Dahlonega, Ga.


### EPSOM SALTS FOR THE PAIN IN BURNS.

BY N. F. HOWARD, M. D.,

DAHLONEGA, GA.

On the morning of December 5th, 1893, we were called to see Mr. W. S. Huff. We found him with the palmar surface of both his hands and fingers burned to a blister. His wife's dress had caught fire and he burned his hands extinguishing the fire. When we saw him he had his hands in a bowl of water, syrup and soap, and was suffering intensely.

We gave him one-third grain of morphine and dressed the hands with lime water and oil mixed. The pain continued and at the end of thirty minutes we administered another dose of morphine; but there was no relief—the suffering seemed to increase. His skin became cold and was covered with perspiration, and his agony was so extreme that we feared lockjaw. In this immergency we decided to try a remedy my wife used with success in a burn twenty-five years ago. We dissolved one pound of epsom salts in two quarts of water and immersed the hands of Mr. H. in this solution. Almost instantly he was free from pain. He was now so comfortable—talking and laughing with friends—that it was not easy to realize that he was the same man who was in so much distress a few moments before. We let the hands remain in the preparation one hour.

When they were put into the solution they were red, swelled, hot and painful. When taken out the redness, heat, pain and swelling were about gone. Yet the remedy was so simple and safe.

The hands were dressed as before—with lime water and oil mixed—dusting oxide of zinc upon the raw spots where the skin was off.

The hands were soon well with this light dressing.

In one hour from the time the hands were removed from the solution and dressed, Mr. H. was at the courthouse attending to business.

This case is so remarkable, especially when taken along with the one that Mrs. H. used this remedy in with such happy results, and of which we had lost sight of till this case came up, that we thought it should be made public.

We are now well satisfied that in two cases salts relieved the local pain of burns—it might be said instantly.

